# Short-Term Fluctuations in Air Pollution and Asthma in Scania, Sweden. Is the Association Modified by Long-Term Concentrations?

**DOI:** 10.1371/journal.pone.0166614

**Published:** 2016-11-18

**Authors:** Tahir Taj, Emilie Stroh, Daniel Oudin Åström, Kristina Jakobsson, Anna Oudin

**Affiliations:** 1 Department of Occupational and Environmental Medicine, Lund University, Lund, Sweden; 2 Centre for Primary Health Care Research, Department of Clinical Science, Malmö, Lund University, Lund, Sweden; 3 Department of Occupational and Environmental Medicine, Umeå University, Umeå, Sweden; Telethon Institute for Child Health Research, AUSTRALIA

## Abstract

**Background and aims:**

Asthma is one of the most common respiratory diseases in the world. Research has shown that temporal increases in air pollution concentrations can aggravate asthma symptoms. The aim of this study was to assess whether individuals living in areas with higher air pollution concentrations responded differently to short-term temporal exposure to air pollution than those living in lower air pollution areas.

**Method:**

The study was designed as a case-crossover study in Scania, Sweden. Outcome data was visits to primary health care clinics with asthma as the main complaint during the years 2007 to 2010. Nitrogen dioxide levels were obtained from 21 different air pollution monitoring stations. Short-term exposure was defined as the average concentration four days prior to the visit. Data was pooled for areas above and below a two-year average NO2 concentration of 10 μg/m3, dispersion modelled with an emission database.

**Results:**

The short-term association between NO2 and asthma visits seemed stronger in areas with NO2 levels below 10 μg/m3, with an odds ratio (OR) of 1.15 (95% confidence interval (CI): 1.08–1.23) associated with a 10 μg/m3 increase in NO_2_ compared to areas above 10 μg/m3 NO2 levels, where corresponding OR of 1.09 (95% CI: 1.02–1.17). However, this difference was not statistically significant. (p = 0.13)

**Conclusions:**

The study provided some evidence, although not statistically significant, that short-term associations between air pollution and asthma may depend on background air pollution levels. However, we cannot rule out that the association is due to other spatially dependent factors in Scania. The study should be reproduced in other study areas.

## Introduction

Asthma is one of the most common chronic diseases among adolescents and middle-aged adults, and has become a major public health problem worldwide over the last few decades [1]. The prevalence of asthma in Sweden has stabilized during the last few decades, but is still one of the main public health concerns [2]. In a large population-based study in Sweden, the prevalence of physician diagnosed asthma was 8.3% Of these, 70% were actively using asthma medication for symptom control [3].

A recent multicentre European study has shown that people growing up in rural areas close to livestock had a significantly lower prevalence of asthma compared to those growing up in urban areas [4]. Studies from Africa [5] and South America [6] have also reported similar findings. The main explanation for this difference is likely to be that the evolution of the Western lifestyle has resulted in relatively limited exposure to infectious agents during childhood [7], which expedite atopy by affecting the overall array of commensals and pathogens [8]. Another major difference between urban and rural areas, in terms of asthma risk factors, are air pollution concentrations.

Associations between air pollution and asthma are fairly well studied; air pollutants trigger inflammatory response and can act as strong bronchoconstrictors, and thereby exacerbate asthma symptoms [9, 10]. Air pollution levels are associated with increased health care visits due to asthma [11], for both emergency care and hospital admission [11] as well as for anti-asthmatic prescription [12]. However, it is more uncertain whether long-term exposure to air pollution is a cause of incident asthma [13–17].

Studies combining short-term and long-term exposure to air pollution are almost entirely lacking. A combined short-term and long-term approach is necessary to investigate if the effect of exposure to elevated levels of air pollutants shortly before an episode of exacerbation of respiratory problems is also dependent on long-term (several years) exposure levels [18]. Indeed, it may be that the burden of asthma exacerbations attributable to air pollution relates not to the triggering per se, but to air pollution increasing the pool of subjects with chronic obstructive diseases [19]. There is therefore a need to understand vulnerability to short-time variations in pollutant levels with respect to long-term exposure levels. The aim of this study was to assess whether individuals living in areas with higher long-term air pollution levels respond differently to a short-term increase in air pollution concentrations than those living in areas with lower long-term air pollution concentrations. The study was performed in an area where pollution levels generally were below or at current air quality guideline levels.

## Methodology

### Study area and population

**Fig 1:**
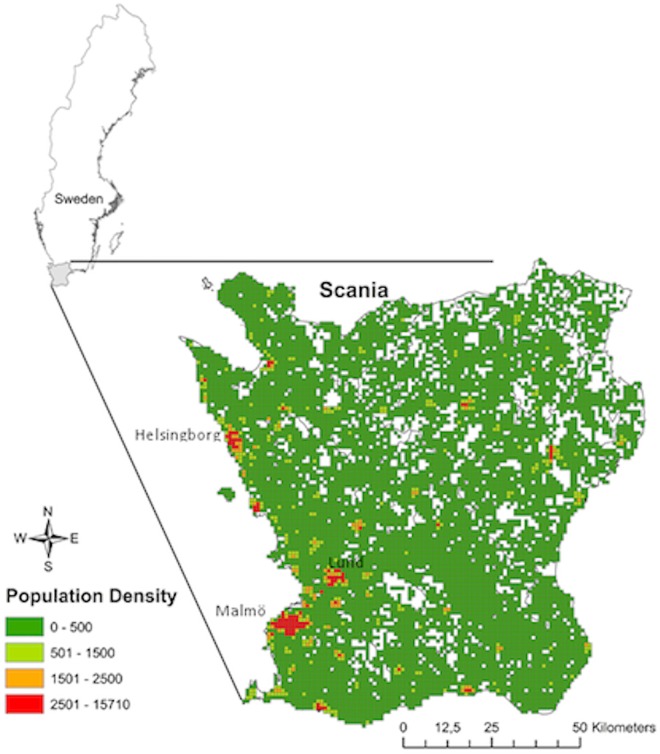
Study area Scania population density map.

The study was performed in the county Scania, Sweden “Fig 1”. Scania is the southernmost county of Sweden and comprises 33 municipalities with a total population of about 1.25 million people. The population density is highest on the west coast, with its three main cities (Malmö, Helsingborg and Lund).

### Data

#### Health care registry

Primary health care data was obtained from the Scania health care register database. In Scania, health care registers, along with data relating to inpatient and emergency visits, also include records of primary health care visits.

Primary health care records from 2007 to 2010 were extracted from the main dataset using ICD 10 codes for asthma illness (J45 codes) and a Swedish translation of ICD 10 codes (J45-p). A total of 20,909 first asthma visits during the study period for individuals living in Scania were extracted for 123 primary health care centres (PHCCs). It is important to note that the outcome used was thus not necessarily the first ever asthma visit, but the first asthma visit during the study period.

**Fig 2:**
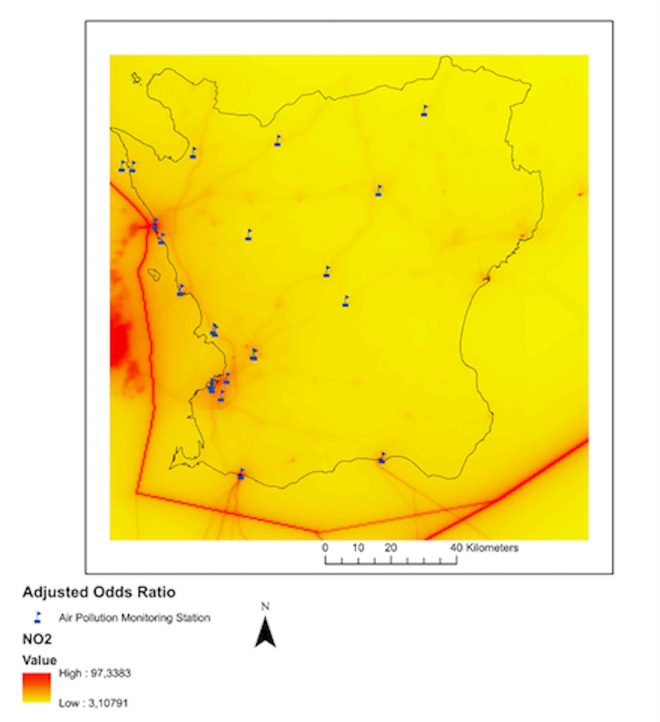
Dispersion model NO_2_ levels and sites of air pollution monitoring stations.

There were nine PHCCs with fewer than 30 visits (a total of 267 visits) that were excluded from the analysis “Fig 2” due to lack of statistical power.

#### Air pollution monitoring stations and dispersion model data

**Fig 3:**
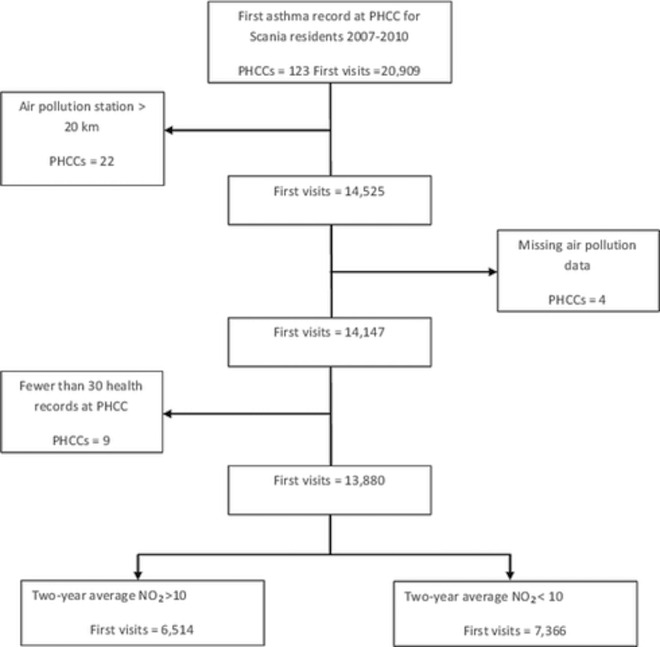
Flow chart showing final sample selection steps.

For this study, nitrogen dioxide (NO_2_) was used as a proxy for air pollution exposure, as a number of studies have shown that NO_2_ is a good indicator of traffic-related air pollution [20–22]. We had access to hourly values from 21 air pollution monitoring stations in different parts of Scania “Fig 3”. For all monitoring stations, geographical coordinates were obtained. We used only data from urban background air pollution monitoring stations to assign short-term air pollution exposure.

NO_2_ levels for each PHCC were assigned based on proximity to the air pollution monitoring station (ranked based on distance from the clinic). PHCCs with no air pollution station within a 20 km radius were excluded from the study (N = 22) (excluding 6,384 asthma visits). We defined the mean air pollution concentration for each PHCs as the (arithmetic) mean concentration for the exposure period for the closest air pollution monitor. We defined the global mean as the (arithmetic) mean concentration from all air pollution monitoring stations in Scania for the exposure time period.

Short-term air pollution exposure was defined as the average NO_2_ level during the same day as the PHCC visit as well as three days prior to the visit. A GIS-based dispersion model together with an emission database was used to model long-term concentrations of NO_2_ at the PHCC [23]. Long-term NO_2_ exposure was defined as the average NO_2_ level at the PHCC two years prior to the visit.

### Statistical analysis

#### Study design

A time stratified case-crossover study design was used [24]. This design is used extensively in environmental studies to analyse the short-term effects of environmental exposure such as air pollutants [25] and temperature [26, 27] on health. This study design compares the exposure prior to a health event with control periods that can be either prior to or after the event. The same individual acts as his or her own control within a short time span, which is why this design adjusts by design for non-time varying confounders at individual level such as gender, education and socioeconomic factors [28]. For each visit, four control periods matched by day of the week were calculated, two prior to the visit and two after, using the same air pollution monitoring station. The total number of first asthma visits selected for final analysis was 13,880 at 88 PHCCs. Detailed descriptions of first visit selection are explained in “Fig 2”.

Model 1 was unadjusted with only NO_2_ as a covariate, model 2 was adjusted for temperature, Humidity and rainfall with smooth functions (natural cubic splines) with 3 degrees of freedom. PM_10_ was additionally adjusted for in model 3, O_3_ in model 4 and SO_2_ in model 5. Model checks were performed using residual deviance, i.e. plotting residuals versus fitted values and checking R^2^ and calculating AIC. Furthermore, sensitivity analysis was performed using the mean pollutant level for each PHCC instead of the global mean and when not excluding data based on number of visits and gender.

All PHCCs were grouped in to two categories of NO_2,_ above and below 10 μg/m^3^ using long term air pollution exposure i.e two year prior to visit. We also calculated a relative risk ratio between the exposure groups and evaluated the statistical significance of the derived Z-score and corresponding p-value [29].

PostgreSQL 9.1.3 relational database [30] was used for identifying the first asthma visit and calculating short- and long-term NO_2_ levels for each visit. Data analyses were performed with R version 3.2[31].

### Ethics statement

Our request for primary health care data was granted after formal scrutiny at the Region Skånes Health Care Databases. In accordance with Swedish law and regulations, we did not seek permission at the Regional Ethical Board at Lund University, since the data granted to us had no personal identification numbers and very limited individual information. It was impossible to identify any individual from our data.

## Results

The total number of first asthma visits during the study period was 13,880 at 88 PHCC. The mean age of the patients was 46 years (SD: 24), and 7,161 (56.3%) were female. The mean daily first asthma visits for each PHCC ranged from 3 to 319. Descriptive data on daily weather and air pollution levels is given in Table 1.

**Table 1:** Descriptive data on daily weather, air pollution in Scania, Sweden, 2007–2010.

Variable	Minimum	25%	Median	75%	Maximum	Mean ± SD
**PM**_10_ μg/m3	4.1	12.7	15.6	19.8	46.4	16.7 ± 6.2
**PM**_2.5_ μg/m3	2.9	7.6	9.6	13.0	57.0	10.9 ± 5.5
**NO**_2_ μg/m3	0.7	9.7	14.4	19.2	60.6	14.3 ± 8.1
**O**_3_ μg/m3	20.7	46.3	55.9	65.1	93.2	55.3 ± 13.1
**SO**_2_ μg/m3	1.0	2.1	2.6	3.2	6.8	2.7 ± 0.9
**Temperature** °C	-7.0	4.0	8.3	14.1	25.3	8.8 ± 6.5
**Humidity** %	46.1	67.4	76.0	82.3	94.0	74.2 ± 10.0
**Rain** mm	0.0	0.0	0.7	2.5	27.4	1.5 ± 2.8
Asthma visits	1	9	13	17	38	13 ± 6.6

Long-term NO_2_ levels at PHCCs towards the west coast were higher compared to the east coast “Fig 3”. [In S1 Table and S2 Table, the correlations between pollutants and climate data are reported.] Tables 2 and 3 show pooled unadjusted and adjusted odds ratios for PHCC above and below 10NO_2_ μg/m^3^.

**Table 2:** Pooled odds ratios (ORs)for Asthma visit with their 95% confidence intervals (CIs) for areas with NO_2_ below 10 μg/m3.

	OR	95% CI	P value
Model 1 NO_2_ only	1.15	1.08–1.23	<0.001
Model 2 temp hum rainfall added	1.15	1.08–1.23	<0.001
Model 3 PM_10_ added	1.13	1.18–1.07	<0.001
Model 4 O_3_ added	1.13	1.18–1.07	<0.001
Model 5 SO_2_ added	1.12	1.17–1.07	<0.001

Associations are shown as ORs and their 95% CIs for each pollutant

Model 1 effect of NO_2_ without adjustment

Model 2 effect of NO_2_ with adjustment temperature, humidity and rainfall

Model 3 effect of NO_2_ with adjustment temperature, humidity, rainfall and PM_10_

Model 4 effect of NO_2_ with adjustment temperature, humidity, rainfall, PM_10_ and O_3_

Model 5 effect of NO_2_ with adjustment temperature, humidity, rainfall, PM_10_, O_3_ and SO_2_

**Table 3:** Pooled odds ratios (ORs) for Asthma visit with their 95% confidence intervals (CIs) for areas with NO_2_ above 10 μg/m3.

	Odds Ratio	95% CI	P value
Model 1 NO_2_ only	1.10	1.03–1.18	<0.01
Model 2 temp hum rainfall added	1.09	1.02–1.15	<0.01
Model 3 PM_10_ added	1.10	1.02–1.18	<0.01
Model 4 O_3_ added	1.09	1.03–1.18	<0.01
Model 5 SO_2_ added	1.09	1.02–1.17	<0.01

Associations are shown as ORs and their 95% CIs for each pollutant.

Model 1 effect of NO_2_ without adjustment

Model 2 effect of NO_2_ with adjustment temperature, humidity and rainfall

Model 3 effect of NO_2_ with adjustment temperature, humidity, rainfall and PM_10_

Model 4 effect of NO_2_ with adjustment temperature, humidity, rainfall, PM_10_ and O_3_

Model 5 effect of NO_2_ with adjustment temperature, humidity, rainfall, PM_10_, O_3_ and SO_2_

**Fig 4:**
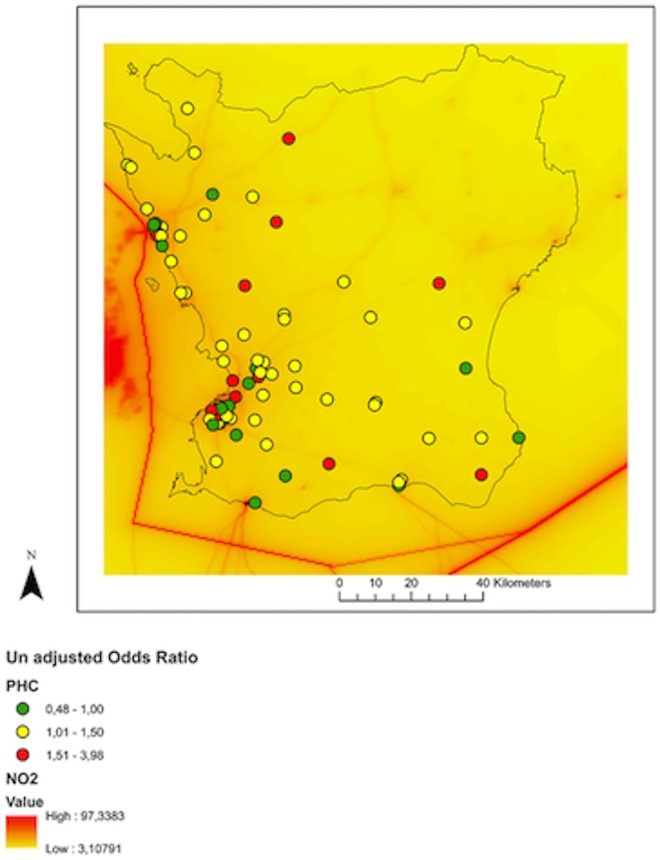
Unadjusted odds ratio at PHCC level.

**Fig 5:**
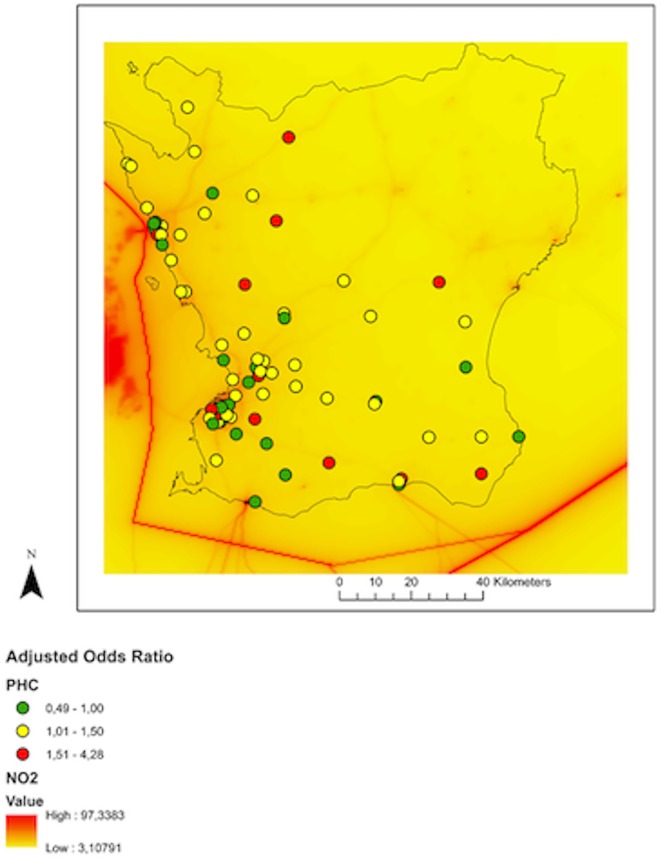
Adjusted odds ratio at PHCC level.

Odds ratios for each PHCC are plotted in “Figs 4 and 5” and given in Table 4 and also in “S1 Fig and S2 Fig”. A pooled odds ratio adjusting for temperature, humidity, rainfall and other pollutants was calculated for PHCCs with long-term NO_2_ levels above and below 10 μg/m^3^. Tables 2 and 3 show detailed steps of the model for pooled analysis. The pooled odds ratios for PHCCs with long term lower NO_2_ levels was 1.15 (95% confidence interval (CI): 1.08–1.23), whereas the pooled odds ratio for PHCCs with long term higher NO_2_ levels was 1.09 (95% CI: 1.02–1.17). The difference between the pooled odds ratios was not statistically significant (p = 0.13).

**Table 4:** Odds ratios (ORs)with 95% confidence intervals (CIs) for Asthma visits.

PHCC ID	Odds ratio	Lower limit	Upper limit
12	0.89	0.64	1.23
17	103	0.69	1.53
25	1.20	0.74	1.94
27	0.86	0.51	1.48
32	1.15	0.56	2.37
34	NA	NA	NA
40	0.7	0.47	1.05
41	1.64	0.77	3.51
44	0.49	0.21	1.14
46	0.97	0.51	1.85
50	2.02	0.85	4.81
51	1.11	0.58	2.13
53	0.99	0.47	2.06
54	1.03	0.43	2.51
55	0.91	0.64	1.29
63	1.50	1.01	2.22
64	0.98	0.60	1.60
65	1.28	0.95	1.74
66	1.12	0.80	1.57
67	1.42	0.99	2.04
68	1.01	0.64	1.58
69	1.36	0.78	2.37
70	1.33	0.99	1.78
71	0.75	0.51	1.12
72	1.08	0.69	1.68
73	1.25	0.77	2.03
74	1.59	1.06	2.36
75	1.16	0.86	1.58
76	1.33	0.97	1.82
78	1.25	0.79	1.98
79	1.67	1.16	2.39
80	1.31	0.86	1.99
81	1.02	0.71	1.48
82	1.06	0.72	1.56
83	1.20	0.91	1.55
84	1.00	0.74	1.35
85	NA		
87	NA		
90	NA		
91	1.10	0.61	2.01
92	NA		
93	3.62	0.75	17.41
96	NA		
97	NA		
98	NA		
99	NA		
103	0.80	0.47	1.36
104	1.01	0.59	1.73
105	0.95	0.64	1.42
106	0.74	0.49	1.10
107	1.35	0.88	2.07
108	1.03	0.66	1.59
109	1.57	0.99	2.48
110	1.52	0.89	2.58
111	1.17	0.74	1.85
112	0.97	0.62	1.51
113	1.06	0.71	1.59
114	0.87	0.61	1.23
115	0.96	0.66	1.41
116	1.61	0.98	2.7
117	1.15	0.74	1.81
118	0.69	0.36	1.32
119	0.96	0.64	1.45
120	1.67	0.95	2.94
122	0.93	0.57	1.52
123	1.50	0.98	2.29
125	1.05	0.68	1.60
126	1.69	0.46	6.30
128	1.14	0.52	2.5
130	4.28	0.77	23.9
132	1.07	0.56	2.04
134	1.41	0.75	2.67
135	1.17	0.72	1.91
136	1.13	0.78	1.63
137	1.54	0.89	2.66
138	1.25	0.77	2.04
139	1.31	0.92	1.87
140	1.17	0.69	2.01
141	0.83	0.59	1.18
142	1.22	0.71	2.10
143	0.67	0.41	1.10
144	0.90	0.53	1.52
145	0.72	0.41	1.25
146	1.41	1.03	1.95
147	1.67	1.10	2.53
148	1.18	0.82	1.71
149	1.36	0.95	1.95
150	0.99	0.70	1.41

Model adjusted for climate variable, PM_10_, O_3_ and SO_2_

## Discussion

There was an association between short term increased in air pollution concentration and daily asthma visits. The association seemed stronger in areas with a lower long-term air pollution concentration then in areas with higher concentration. It is important to note that the difference was not statistically significant, and that there are several possible explanations for our finding.

We are not aware of previous studies investigating if long-term air pollution levels may modify the short-term association between air pollution and asthma. Therefore, we are speculating that one explanation for our finding could be that people living in areas with higher long-term air pollution levels adaptation to some extent to exposure to air pollution. There could also be demographic differences between the populations above and below the cut-off that drive the observed difference. Moreover, the difference we observed was not statistically significant. It would be of interest to carry out similar studies in different study settings.

There is plenty of evidence for air pollution to have acute adverse effects on respiratory symptoms, [32–34] especially in large urban areas where air pollution levels can be rather high, for example in Sao Paolo, Brazil, [35] Hong Kong,[36] Vancouver [37]and Toronto [38], Canada, inner-cities of USA[39] and Copenhagen, Denmark[40]. Effects on incident asthma is more uncertain. For example, a study by Gao Y et al. in 2014 [41] found that girls living in high pollution districts had higher rates of respiratory symptoms as well as an elevated risk of asthma. Another study conducted by Berhane K et al. in 2014 [42] reported a negative association between long-term NO_2_ concentration among asthmatics and lung function; the same study also reported similar results with other criteria pollutants. However, a study conducted by Fuertes E et al. in 2013 [43] failed to find any association between living in high pollutant areas, i.e. expose to traffic-related air pollution, and risk of asthma. In a review by Jie Y et al. [44], the impact of geographical variation on asthma prevalence was studied. In this review they particularly studied the difference in asthma between urban and rural areas. The researchers concluded that asthma symptoms occurred more frequently in urban areas compared to rural areas, and the main explanation they found in their review was a difference in environmental risk factors, with the urban population being more exposed to dust mites and high traffic-related air pollution levels, and that a Western lifestyle contributed to high levels of asthma symptoms [44].

A possible explanation to our finding could be that those who are more susceptible to asthma and other respiratory diseases tend to move away from urban areas, which perhaps partly could explain the discrepant results of studies on long-term air pollution and asthma. However, the findings from previous studies are not directly comparable with our present study since we used primary health care data, which is rather unique (air pollution studies often use cohort data or emergency and hospital admission data). Moreover, our setting is a generally low air pollution setting, unlike other studies. If the results of the present study would be repeated and corroborated by others, they are interesting in the context of understanding the link between air pollution and asthma, which is still only partly understood.

### Strengths and weaknesses

The study has several strengths and weaknesses that should be mentioned. First, we did not have access to participants’ exact residential addresses or workplace addresses, which naturally is a source of exposure measurement error, and is always an issue in time series air pollution studies [45]. We know from previous studies that measured exposure is not even highly correlated with modelled exposure at home [23] for certain. However, when we studied the study subjects’ residential address and where they seek health care, we found that more than 99% of the individuals visited a PHCC in the same municipality where they were registered as being resident “S3 Table–S8 Table”. We also studied the change in residential address for our study participants over the study period, and we found that 90% of the participants had not changed their residence over the entire study period; 1,64% had a residential address different from the PHCC commune they visited, 3,52% had a residential address different from the PHCC commune one year prior to the visit and 4,54% had a residential address different from the PHCC commune they visited two years prior to visit. Detailed yearly statistics are given in “S9 Table”. This indicates that exposure misclassification caused by the fact that patients seek healthcare outside their residential municipality is rather limited.

One strength of this study is that air pollution exposure was assigned using 21 air pollution monitoring stations spread across the county “Fig 2”. To improve exposure assessment, all PHCCs with no air pollution monitoring station within the nearest 20 km were excluded from the study, which reduced the total number of PHCCs analysed in the final model but probably added accuracy to the exposure assessment. Another strength of the study is the utilization of modelled NO_2_ data for accessing long-term air pollution exposure. The model used has been validated in a prior study [46]. However, we did not have enough information to conduct enough sensitivity analyses to rule out the possibility that our findings could be due to underlying differences in, for example, demographic differences between populations below and above the cut-off background air pollution concentrations. The main strength of the study is the rather unique outcome data, where we were able to use primary health care data on a large scale. Sweden is one of very few countries where primary health care data registers exist and are well organized. Use of PHCC data provides a rare opportunity to study the effects of air pollution on health care visits, which are very different to emergency and inpatient visits. Primary health care is usually the very first health care contact in the disease process. It may therefore be more accurate in associating exposure with disease outcome as it also reflects the initiation of the disease process.

## Conclusion

Our results may suggest that short-term associations between daily fluctuations in air pollution concentrations and primary health care visits for asthma differ depending on background air pollution levels. Further research is needed that explain relationship between air pollution and other spatially determinant covariates for asthma.

## Supporting Information

S1 Fig Unadjusted odds ratio at PHCC level. (DOCX)

S2 Fig Adjusted odds ratio at PHCC level. (DOCX)

S1 Table Correlation coefficients among concentration of air pollutants in areas with NO2 above 10 μg/m3. (DOCX)

S2 Table Correlation coefficients among concentration of air pollutants in areas with NO2 below 10 μg/m3. (DOCX)

S3 Table Yearly visits and percentage of visit outside residential commune same year one year and two year prior to health visits for study Participants. (DOCX)

S4 Table Commune wise health care visits and percentage of visit outside residential commune for Year 2005. (DOCX)

S5 Table Commune wise health care visits and percentage of visit outside residential commune for Year 2006. (DOCX)

S6 Table Commune wise health care visits and percentage of visit outside residential commune for Year 2007. (DOCX)

S7 Table Commune wise health care visits and percentage of visit outside residential commune for Year 2008. (DOCX)

S8 Table Commune wise health care visits and percentage of visit outside residential commune for Year 2009. (DOCX)

S9 Table Commune wise health care visits and percentage of visit outside residential. (DOCX)
