# Supplementary figures and images for: Short-Term Fluctuations in Air Pollution and Asthma in Scania, Sweden. Is the Association Modified by Long-Term Concentrations?
Source: PLoS One. 2016 Nov 18;11(11):e0166614. doi: 10.1371/journal.pone.0166614 (PMC5115756; doi:10.1371/journal.pone.0166614)

S1 Figure Unadjusted odds ratio at PHCC level
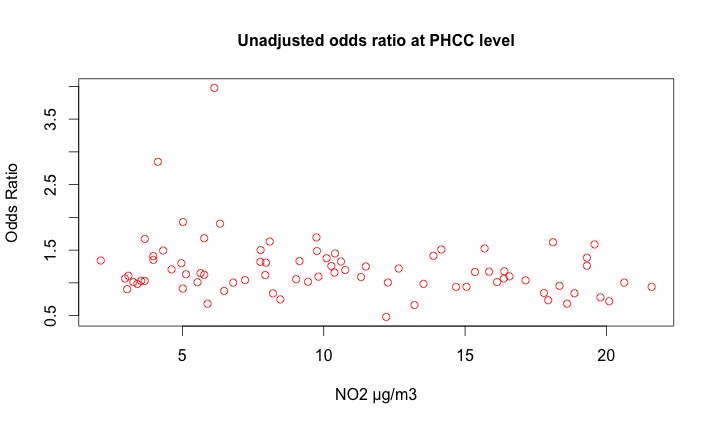

Supplement: S1 Fig — (DOCX) [file pone.0166614.s001.docx]

S2 Figure Adjusted odds ratio at PHCC level
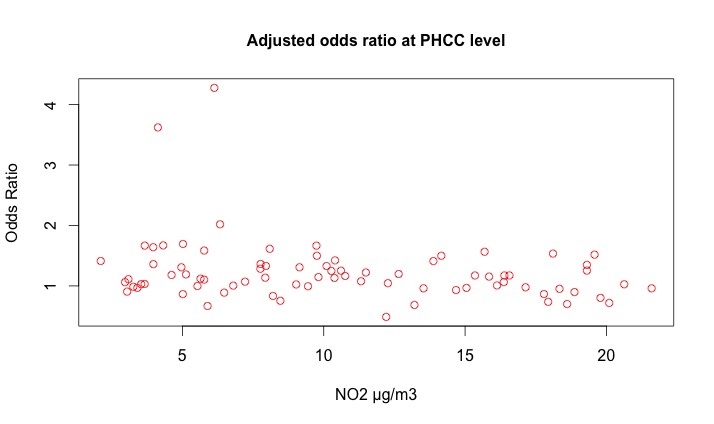

Supplement: S2 Fig — (DOCX) [file pone.0166614.s002.docx]
