# Supplementary material for: Short-Term Fluctuations in Air Pollution and Asthma in Scania, Sweden. Is the Association Modified by Long-Term Concentrations?
Source: PLoS One. 2016 Nov 18;11(11):e0166614. doi: 10.1371/journal.pone.0166614 (PMC5115756; doi:10.1371/journal.pone.0166614)
Supplement: S2 Table — (DOCX) [file pone.0166614.s004.docx]

|  | NO_2_ | PM_10_ | O_3_ | SO_2_ |
| --- | --- | --- | --- | --- |
| NO_2_ | 1 |  |  |  |
| PM_10_ | 0.14 | 1 |  |  |
| O_3_ | -0.01 | 0.17 | 1 |  |
| SO_2_ | 0.09 | 0.53 | 0.04 | 1 |
| Adjusted for temperature, humidity and rainfall | | | | |

S2 Table Correlation coefficients among concentration of air pollutants in areas with NO_2_ below 10 µg/m3
