# Supplementary material for: Short-Term Fluctuations in Air Pollution and Asthma in Scania, Sweden. Is the Association Modified by Long-Term Concentrations?
Source: PLoS One. 2016 Nov 18;11(11):e0166614. doi: 10.1371/journal.pone.0166614 (PMC5115756; doi:10.1371/journal.pone.0166614)
Supplement: S3 Table — (DOCX) [file pone.0166614.s005.docx]

|  |  | **No Change in Residential Address** | **Residential Address change over time** | | |
| --- | --- | --- | --- | --- | --- |
| **Year** | **Total Visits n** | **n (%)** | **Same Year  n (%)** | **One Year Before Visit n (%)** | **Two Year Before Visit n (%)** |
| **2007** | 4,089 | 3,779 (92,4) | 63 (1,5) | 63 (1,5) | 184 (4,5) |
| **2008** | 2,813 | 2,579 (91,7) | 48 (1,7) | 47 (1,7) | 139 (4,9) |
| **2009** | 3,577 | 3,206 (89,6) | 62 (1,7) | 154 (4,3) | 155 (4,3) |
| **1010** | 3,400 | 2,968 (87,3) | 55 (1,6) | 225 (6,6) | 152 (4,5) |
| **Total** | 13,880 | 12,533 (90,3) | 228 (1,6) | 489 (3,5) | 630 (4,5) |

S3 Table Yearly visits and percentage of visit outside residential commune same year one year and two year prior to health visits for study Participants
