# Supplementary material for: Short-Term Fluctuations in Air Pollution and Asthma in Scania, Sweden. Is the Association Modified by Long-Term Concentrations?
Source: PLoS One. 2016 Nov 18;11(11):e0166614. doi: 10.1371/journal.pone.0166614 (PMC5115756; doi:10.1371/journal.pone.0166614)
Supplement: S4 Table — (DOCX) [file pone.0166614.s006.docx]

| **Commune Code** | **Visits in same Commune**  **as Residential Address** | **Visits in different Commune**  **as Residential Address** | **Total Visits** | **% Visits different Commune**  **as Residential Address** | **% Visits same Commune**  **as Residential Address** |
| --- | --- | --- | --- | --- | --- |
|  |  |  |  |  |  |
| **1214** | 16234 | 390 | 16624 | 2,35 | 97,65 |
| **1230** | 27465 | 444 | 27909 | 1,59 | 98,41 |
| **1231** | 24040 | 668 | 24708 | 2,70 | 97,30 |
| **1233** | 30136 | 473 | 30609 | 1,55 | 98,45 |
| **1256** | 20363 | 300 | 20663 | 1,45 | 98,55 |
| **1257** | 9628 | 87 | 9715 | 0,90 | 99,10 |
| **1260** | 16610 | 355 | 16965 | 2,09 | 97,91 |
| **1261** | 31895 | 735 | 32630 | 2,25 | 97,75 |
| **1262** | 22474 | 279 | 22753 | 1,23 | 98,77 |
| **1263** | 18044 | 339 | 18383 | 1,84 | 98,16 |
| **1264** | 15730 | 437 | 16167 | 2,70 | 97,30 |
| **1265** | 20314 | 579 | 20893 | 2,77 | 97,23 |
| **1266** | 14658 | 489 | 15147 | 3,23 | 96,77 |
| **1267** | 16362 | 267 | 16629 | 1,61 | 98,39 |
| **1270** | 19175 | 297 | 19472 | 1,53 | 98,47 |
| **1272** | 8553 | 38 | 8591 | 0,44 | 99,56 |
| **1273** | 11808 | 122 | 11930 | 1,02 | 98,98 |
| **1275** | 10100 | 74 | 10174 | 0,73 | 99,27 |
| **1276** | 18867 | 294 | 19161 | 1,53 | 98,47 |
| **1277** | 18765 | 291 | 19056 | 1,53 | 98,47 |
| **1278** | 13619 | 94 | 13713 | 0,69 | 99,31 |
| **1280** | 264186 | 3437 | 267623 | 1,28 | 98,72 |
| **1281** | 112496 | 1554 | 114050 | 1,36 | 98,64 |
| **1282** | 58874 | 688 | 59562 | 1,16 | 98,84 |
| **1283** | 123645 | 1141 | 124786 | 0,91 | 99,09 |
| **1284** | 18377 | 135 | 18512 | 0,73 | 99,27 |
| **1285** | 40240 | 552 | 40792 | 1,35 | 98,65 |
| **1286** | 35323 | 421 | 35744 | 1,18 | 98,82 |
| **1287** | 43800 | 448 | 44248 | 1,01 | 98,99 |
| **1290** | 95008 | 745 | 95753 | 0,78 | 99,22 |
| **1291** | 35519 | 291 | 35810 | 0,81 | 99,19 |
| **1292** | 36145 | 290 | 36435 | 0,80 | 99,20 |
| **1293** | 62656 | 313 | 62969 | 0,50 | 99,50 |
| **Total** | 1311109 | 1290 | 1312399 | 0,10 | 99,90 |

S4 Table Commune wise health care visits and percentage of visit outside residential commune for Year 2005
