# Supplementary material for: Short-Term Fluctuations in Air Pollution and Asthma in Scania, Sweden. Is the Association Modified by Long-Term Concentrations?
Source: PLoS One. 2016 Nov 18;11(11):e0166614. doi: 10.1371/journal.pone.0166614 (PMC5115756; doi:10.1371/journal.pone.0166614)
Supplement: S5 Table — (DOCX) [file pone.0166614.s007.docx]

| **Commune Code** | **Visits in same Commune**  **as Residential Address** | **Visits in different Commune**  **as Residential Address** | **Total Visits** | **% Visits different Commune**  **as Residential Address** | **% Visits same Commune**  **as Residential Address** |
| --- | --- | --- | --- | --- | --- |
| **1214** | 15676 | 257 | 15933 | 1,61 | 98,39 |
| **1230** | 24755 | 435 | 25190 | 1,73 | 98,27 |
| **1231** | 24734 | 329 | 25063 | 1,31 | 98,69 |
| **1233** | 30916 | 301 | 31217 | 0,96 | 99,04 |
| **1256** | 20428 | 535 | 20963 | 2,55 | 97,45 |
| **1257** | 10261 | 170 | 10431 | 1,63 | 98,37 |
| **1260** | 17446 | 270 | 17716 | 1,52 | 98,48 |
| **1261** | 32042 | 392 | 32434 | 1,21 | 98,79 |
| **1262** | 23920 | 287 | 24207 | 1,19 | 98,81 |
| **1263** | 21245 | 594 | 21839 | 2,72 | 97,28 |
| **1264** | 17059 | 372 | 17431 | 2,13 | 97,87 |
| **1265** | 20427 | 443 | 20870 | 2,12 | 97,88 |
| **1266** | 15361 | 243 | 15604 | 1,56 | 98,44 |
| **1267** | 17478 | 339 | 17817 | 1,90 | 98,10 |
| **1270** | 20468 | 499 | 20967 | 2,38 | 97,62 |
| **1272** | 8855 | 100 | 8955 | 1,12 | 98,88 |
| **1273** | 11909 | 108 | 12017 | 0,90 | 99,10 |
| **1275** | 9843 | 203 | 10046 | 2,02 | 97,98 |
| **1276** | 18889 | 382 | 19271 | 1,98 | 98,02 |
| **1277** | 20354 | 301 | 20655 | 1,46 | 98,54 |
| **1278** | 13569 | 165 | 13734 | 1,20 | 98,80 |
| **1280** | 285755 | 3438 | 289193 | 1,19 | 98,81 |
| **1281** | 117793 | 1648 | 119441 | 1,38 | 98,62 |
| **1282** | 62624 | 651 | 63275 | 1,03 | 98,97 |
| **1283** | 134203 | 1433 | 135636 | 1,06 | 98,94 |
| **1284** | 20060 | 230 | 20290 | 1,13 | 98,87 |
| **1285** | 43428 | 1006 | 44434 | 2,26 | 97,74 |
| **1286** | 37021 | 323 | 37344 | 0,86 | 99,14 |
| **1287** | 48650 | 603 | 49253 | 1,22 | 98,78 |
| **1290** | 96906 | 603 | 97509 | 0,62 | 99,38 |
| **1291** | 36979 | 472 | 37451 | 1,26 | 98,74 |
| **1292** | 36606 | 348 | 36954 | 0,94 | 99,06 |
| **1293** | 64151 | 442 | 64593 | 0,68 | 99,32 |
| **Total** | 1379811 | 17922 | 1397733 | 1,28 | 98,72 |

S5 Table Commune wise health care visits and percentage of visit outside residential commune for Year 2006
