# Supplementary material for: Short-Term Fluctuations in Air Pollution and Asthma in Scania, Sweden. Is the Association Modified by Long-Term Concentrations?
Source: PLoS One. 2016 Nov 18;11(11):e0166614. doi: 10.1371/journal.pone.0166614 (PMC5115756; doi:10.1371/journal.pone.0166614)
Supplement: S6 Table — (DOCX) [file pone.0166614.s008.docx]

| **Commune Code** | **Visits in same Commune**  **as Residential Address** | **Visits in different Commune**  **as Residential Address** | **Total Visits** | **% Visits different Commune**  **as Residential Address** | **% Visits same Commune**  **as Residential Address** |
| --- | --- | --- | --- | --- | --- |
|  |  |  |  |  |  |
| **1214** | 17417 | 454 | 17871 | 2,54 | 97,46 |
| **1230** | 25864 | 532 | 26396 | 2,02 | 97,98 |
| **1231** | 24776 | 451 | 25227 | 1,79 | 98,21 |
| **1233** | 32879 | 282 | 33161 | 0,85 | 99,15 |
| **1256** | 20286 | 376 | 20662 | 1,82 | 98,18 |
| **1257** | 10449 | 201 | 10650 | 1,89 | 98,11 |
| **1260** | 17153 | 474 | 17627 | 2,69 | 97,31 |
| **1261** | 33330 | 697 | 34027 | 2,05 | 97,95 |
| **1262** | 23946 | 418 | 24364 | 1,72 | 98,28 |
| **1263** | 21486 | 441 | 21927 | 2,01 | 97,99 |
| **1264** | 17367 | 409 | 17776 | 2,30 | 97,70 |
| **1265** | 22006 | 606 | 22612 | 2,68 | 97,32 |
| **1266** | 15273 | 437 | 15710 | 2,78 | 97,22 |
| **1267** | 16845 | 280 | 17125 | 1,64 | 98,36 |
| **1270** | 20808 | 369 | 21177 | 1,74 | 98,26 |
| **1272** | 9200 | 82 | 9282 | 0,88 | 99,12 |
| **1273** | 11814 | 102 | 11916 | 0,86 | 99,14 |
| **1275** | 10519 | 175 | 10694 | 1,64 | 98,36 |
| **1276** | 20423 | 429 | 20852 | 2,06 | 97,94 |
| **1277** | 21873 | 319 | 22192 | 1,44 | 98,56 |
| **1278** | 13431 | 246 | 13677 | 1,80 | 98,20 |
| **1280** | 288841 | 4170 | 293011 | 1,42 | 98,58 |
| **1281** | 119195 | 1624 | 120819 | 1,34 | 98,66 |
| **1282** | 63616 | 826 | 64442 | 1,28 | 98,72 |
| **1283** | 136445 | 1206 | 137651 | 0,88 | 99,12 |
| **1284** | 20820 | 191 | 21011 | 0,91 | 99,09 |
| **1285** | 43620 | 721 | 44341 | 1,63 | 98,37 |
| **1286** | 39022 | 381 | 39403 | 0,97 | 99,03 |
| **1287** | 54485 | 396 | 54881 | 0,72 | 99,28 |
| **1290** | 98788 | 905 | 99693 | 0,91 | 99,09 |
| **1291** | 37488 | 554 | 38042 | 1,46 | 98,54 |
| **1292** | 37080 | 535 | 37615 | 1,42 | 98,58 |
| **1293** | 66762 | 509 | 67271 | 0,76 | 99,24 |
| **Total** | 1413307 | 19798 | 1433105 | 1,38 | 98,62 |

S6 Table Commune wise health care visits and percentage of visit outside residential commune for Year 2007
