# Supplementary material for: Short-Term Fluctuations in Air Pollution and Asthma in Scania, Sweden. Is the Association Modified by Long-Term Concentrations?
Source: PLoS One. 2016 Nov 18;11(11):e0166614. doi: 10.1371/journal.pone.0166614 (PMC5115756; doi:10.1371/journal.pone.0166614)
Supplement: S7 Table — (DOCX) [file pone.0166614.s009.docx]

| **Commune Code** | **Visits in same Commune**  **as Residential Address** | **Visits in different Commune**  **as Residential Address** | **Total Visits** | **% Visits different Commune**  **as Residential Address** | **% Visits same Commune**  **as Residential Address** |
| --- | --- | --- | --- | --- | --- |
|  |  |  |  |  |  |
| **1214** | 17296 | 436 | 17732 | 2,46 | 97,54 |
| **1230** | 26778 | 605 | 27383 | 2,21 | 97,79 |
| **1231** | 26928 | 324 | 27252 | 1,19 | 98,81 |
| **1233** | 34161 | 315 | 34476 | 0,91 | 99,09 |
| **1256** | 20259 | 199 | 20458 | 0,97 | 99,03 |
| **1257** | 9434 | 308 | 9742 | 3,16 | 96,84 |
| **1260** | 17776 | 620 | 18396 | 3,37 | 96,63 |
| **1261** | 35368 | 502 | 35870 | 1,40 | 98,60 |
| **1262** | 25508 | 268 | 25776 | 1,04 | 98,96 |
| **1263** | 20135 | 271 | 20406 | 1,33 | 98,67 |
| **1264** | 18236 | 384 | 18620 | 2,06 | 97,94 |
| **1265** | 23106 | 491 | 23597 | 2,08 | 97,92 |
| **1266** | 15290 | 233 | 15523 | 1,50 | 98,50 |
| **1267** | 17086 | 354 | 17440 | 2,03 | 97,97 |
| **1270** | 22196 | 453 | 22649 | 2,00 | 98,00 |
| **1272** | 8486 | 114 | 8600 | 1,33 | 98,67 |
| **1273** | 12882 | 250 | 13132 | 1,90 | 98,10 |
| **1275** | 10613 | 166 | 10779 | 1,54 | 98,46 |
| **1276** | 20389 | 233 | 20622 | 1,13 | 98,87 |
| **1277** | 23076 | 330 | 23406 | 1,41 | 98,59 |
| **1278** | 14119 | 127 | 14246 | 0,89 | 99,11 |
| **1280** | 300679 | 3888 | 304567 | 1,28 | 98,72 |
| **1281** | 124747 | 1595 | 126342 | 1,26 | 98,74 |
| **1282** | 64593 | 785 | 65378 | 1,20 | 98,80 |
| **1283** | 137817 | 1591 | 139408 | 1,14 | 98,86 |
| **1284** | 21365 | 299 | 21664 | 1,38 | 98,62 |
| **1285** | 45653 | 764 | 46417 | 1,65 | 98,35 |
| **1286** | 41663 | 445 | 42108 | 1,06 | 98,94 |
| **1287** | 56269 | 688 | 56957 | 1,21 | 98,79 |
| **1290** | 103154 | 842 | 103996 | 0,81 | 99,19 |
| **1291** | 38575 | 353 | 38928 | 0,91 | 99,09 |
| **1292** | 39922 | 296 | 40218 | 0,74 | 99,26 |
| **1293** | 69320 | 586 | 69906 | 0,84 | 99,16 |
| **Total** | 1462879 | 19115 | 1481994 | 1,29 | 98,71 |

S7 Table Commune wise health care visits and percentage of visit outside residential commune for Year 2008
