# Supplementary material for: Short-Term Fluctuations in Air Pollution and Asthma in Scania, Sweden. Is the Association Modified by Long-Term Concentrations?
Source: PLoS One. 2016 Nov 18;11(11):e0166614. doi: 10.1371/journal.pone.0166614 (PMC5115756; doi:10.1371/journal.pone.0166614)
Supplement: S8 Table — (DOCX) [file pone.0166614.s010.docx]

| **Commune Code** | **Visits in same Commune**  **as Residential Address** | **Visits in different Commune**  **as Residential Address** | **Total Visits** | **% Visits different Commune**  **as Residential Address** | **% Visits same Commune**  **as Residential Address** |
| --- | --- | --- | --- | --- | --- |
|  |  |  |  |  |  |
| **1214** | 18702 | 315 | 19017 | 1,66 | 98,34 |
| **1230** | 30015 | 725 | 30740 | 2,36 | 97,64 |
| **1231** | 28683 | 443 | 29126 | 1,52 | 98,48 |
| **1233** | 37154 | 454 | 37608 | 1,21 | 98,79 |
| **1256** | 18483 | 403 | 18886 | 2,13 | 97,87 |
| **1257** | 10742 | 274 | 11016 | 2,49 | 97,51 |
| **1260** | 19540 | 410 | 19950 | 2,06 | 97,94 |
| **1261** | 38115 | 472 | 38587 | 1,22 | 98,78 |
| **1262** | 27172 | 322 | 27494 | 1,17 | 98,83 |
| **1263** | 25758 | 282 | 26040 | 1,08 | 98,92 |
| **1264** | 19772 | 332 | 20104 | 1,65 | 98,35 |
| **1265** | 24783 | 535 | 25318 | 2,11 | 97,89 |
| **1266** | 16191 | 437 | 16628 | 2,63 | 97,37 |
| **1267** | 18553 | 265 | 18818 | 1,41 | 98,59 |
| **1270** | 23678 | 431 | 24109 | 1,79 | 98,21 |
| **1272** | 8767 | 98 | 8865 | 1,11 | 98,89 |
| **1273** | 13863 | 140 | 14003 | 1,00 | 99,00 |
| **1275** | 11654 | 167 | 11821 | 1,41 | 98,59 |
| **1276** | 20580 | 354 | 20934 | 1,69 | 98,31 |
| **1277** | 23893 | 460 | 24353 | 1,89 | 98,11 |
| **1278** | 12590 | 188 | 12778 | 1,47 | 98,53 |
| **1280** | 315826 | 3205 | 319031 | 1,00 | 99,00 |
| **1281** | 130399 | 1829 | 132228 | 1,38 | 98,62 |
| **1282** | 68135 | 811 | 68946 | 1,18 | 98,82 |
| **1283** | 148089 | 1090 | 149179 | 0,73 | 99,27 |
| **1284** | 23116 | 284 | 23400 | 1,21 | 98,79 |
| **1285** | 47913 | 771 | 48684 | 1,58 | 98,42 |
| **1286** | 45912 | 384 | 46296 | 0,83 | 99,17 |
| **1287** | 59427 | 740 | 60167 | 1,23 | 98,77 |
| **1290** | 107563 | 937 | 108500 | 0,86 | 99,14 |
| **1291** | 40911 | 1041 | 41952 | 2,48 | 97,52 |
| **1292** | 41478 | 288 | 41766 | 0,69 | 99,31 |
| **1293** | 73117 | 1209 | 74326 | 1,63 | 98,37 |
| **Total** | 1550574 | 20096 | 1570670 | 1,28 | 98,72 |

S8 Table Commune wise health care visits and percentage of visit outside residential commune for Year 2009
