# Supplementary material for: Short-Term Fluctuations in Air Pollution and Asthma in Scania, Sweden. Is the Association Modified by Long-Term Concentrations?
Source: PLoS One. 2016 Nov 18;11(11):e0166614. doi: 10.1371/journal.pone.0166614 (PMC5115756; doi:10.1371/journal.pone.0166614)
Supplement: S9 Table — (DOCX) [file pone.0166614.s011.docx]

| **Commune Code** | **Visits in same Commune**  **as Residential Address** | **Visits in different Commune**  **as Residential Address** | **Total Visits** | **% Visits different Commune**  **as Residential Address** | **% Visits same Commune**  **as Residential Address** |
| --- | --- | --- | --- | --- | --- |
| **1214** | 17239 | 519 | 17758 | 2,92 | 97,08 |
| **1230** | 28491 | 568 | 29059 | 1,95 | 98,05 |
| **1231** | 25158 | 400 | 25558 | 1,57 | 98,43 |
| **1233** | 35239 | 354 | 35593 | 0,99 | 99,01 |
| **1256** | 17883 | 271 | 18154 | 1,49 | 98,51 |
| **1257** | 11208 | 175 | 11383 | 1,54 | 98,46 |
| **1260** | 17835 | 262 | 18097 | 1,45 | 98,55 |
| **1261** | 36106 | 546 | 36652 | 1,49 | 98,51 |
| **1262** | 25547 | 288 | 25835 | 1,11 | 98,89 |
| **1263** | 27193 | 574 | 27767 | 2,07 | 97,93 |
| **1264** | 19543 | 390 | 19933 | 1,96 | 98,04 |
| **1265** | 24059 | 441 | 24500 | 1,80 | 98,20 |
| **1266** | 17003 | 165 | 17168 | 0,96 | 99,04 |
| **1267** | 18295 | 250 | 18545 | 1,35 | 98,65 |
| **1270** | 22549 | 461 | 23010 | 2,00 | 98,00 |
| **1272** | 8764 | 43 | 8807 | 0,49 | 99,51 |
| **1273** | 13383 | 103 | 13486 | 0,76 | 99,24 |
| **1275** | 11212 | 176 | 11388 | 1,55 | 98,45 |
| **1276** | 19792 | 330 | 20122 | 1,64 | 98,36 |
| **1277** | 22659 | 664 | 23323 | 2,85 | 97,15 |
| **1278** | 11549 | 142 | 11691 | 1,21 | 98,79 |
| **1280** | 306392 | 3106 | 309498 | 1,00 | 99,00 |
| **1281** | 122452 | 1470 | 123922 | 1,19 | 98,81 |
| **1282** | 63228 | 814 | 64042 | 1,27 | 98,73 |
| **1283** | 141493 | 1195 | 142688 | 0,84 | 99,16 |
| **1284** | 22001 | 167 | 22168 | 0,75 | 99,25 |
| **1285** | 44835 | 483 | 45318 | 1,07 | 98,93 |
| **1286** | 45887 | 518 | 46405 | 1,12 | 98,88 |
| **1287** | 55811 | 514 | 56325 | 0,91 | 99,09 |
| **1290** | 102914 | 548 | 103462 | 0,53 | 99,47 |
| **1291** | 40761 | 677 | 41438 | 1,63 | 98,37 |
| **1292** | 39839 | 325 | 40164 | 0,81 | 99,19 |
| **1293** | 71478 | 500 | 71978 | 0,69 | 99,31 |
| **Total** | 1487798 | 17439 | 1505237 | 1,16 | 98,84 |

S9 Table Commune wise health care visits and percentage of visit outside residential commune for Year 2010
